# Cut-off point for diagnosing thoraco-lumbo-pelvic rotation range hypomobility through the leg lateral reach test in chronic low back pain: a cross-sectional study

**DOI:** 10.1590/1516-3180.2024.0500.R1.08092025

**Published:** 2025-12-15

**Authors:** André Pontes-Silva, Aldair Darlan Santos-de-Araújo, Mariana Schamas-Esposel, Aliny da Silva de Araujo, Giovanna Laura Neves Antonio Gaban, Almir Vieira Dibai

**Affiliations:** I Researcher. Postgraduate Program in Physical Therapy, Department of Physical Therapy, Universidade Federal de São Carlos (UFSCar), São Carlos (SP), Brazil.; II PhD student, Postgraduate Program in Physical Therapy, Department of Physical Therapy, Universidade Federal de São Carlos (UFSCar), São Carlos (SP), Brazil.; III Researcher, Department of Anesthesiology, Pain and Intensive Care Medicine, Universidade Federal de São Paulo (Unifesp), São Paulo (SP), Brazil.; IV Researcher, Department of Physical Therapy, Faculdade Santa Terezinha, São Luís (MA), Brazil.; V PhD student, Postgraduate Program in Physical Therapy, Department of Physical Therapy, Universidade Federal de São Carlos (UFSCar), São Carlos (SP), Brazil.; VI Professor, Postgraduate Program in Physical Education, Department of Physical Education, Universidade Federal do Maranhão (UFMA), São Luís (MA), Brazil; Professor, Postgraduate Program in Adult Health, Universidade Federal do Maranhão (UFMA), São Luís (MA), Brazil.

**Keywords:** Chronic pain, Rapid diagnostic tests, ROC curve, Spine, Musculoskeletal pain, Leg lateral reach test, Chronic low back pain, Musculoskeletal system

## Abstract

**BACKGROUND:**

Studies have proposed using the leg lateral reach test (LLRT). However, they did not establish a cut-off point for testing.

**OBJECTIVE:**

To establish a cut-off point for the thoraco-lumbo-pelvic rotation range using the LLRT in patients with chronic low back pain.

**DESIGN AND SETTING:**

Cross-sectional study conducted in Buriticupu, Maranhão, Brazil.

**METHODS:**

In the chronic low back pain group (LBPG, n = 35), we included patients aged 18 to 59 years, of both sexes, with scores ≤ 4 on the Baecke Habitual Physical Activity Questionnaire, body mass indeces ≤ 26 kg/m^2^, disability levels ≥ 3 on the Roland-Morris Disability Questionnaire, and pain levels ≥ 3 on the Numeric Pain Rating Scale. In the healthy control group (HCG, n = 35), the patients had the same characteristics (except for pain and disability). We used receiver operating characteristic curves to check the rate of true versus false positives in different LLRT ranges of motion and found the best LLRT cut-off point using the following mathematical model: (1 – sensitivity)^2^ + (1 – specificity)^2^

**RESULTS:**

The sample was mainly composed of females (HCG = 65.71%; LBPG = 82.85%, P = 0.101), and 68.42% of the characteristics (13 of 19 comparisons between groups) showed a significant difference (P ≤ 0.05), with an effect size ranging from moderate to large (Cohen’s d ≥ 0.5). The cut-off value for ideal sensitivity and specificity was ≤ 82.85 cm.

**CONCLUSION:**

Patients with chronic low back pain and an LLRT range ≤ 82.85 cm have hypomobility regarding thoraco-lumbo-pelvic rotation range.

## INTRODUCTION

 Chronic low back pain is a phenomenon related to pain amplification in the nervous system and is often classified as nonspecific low back pain.^1^ Among 354 medical conditions surveyed in 195 countries, low back pain was the leading cause of lost productivity worldwide and the leading cause of years lived with disability in 126 countries, indicating that low back pain remains poorly understood and undervalued.^2^

 The Psychological Inflexibility in Pain Scale (PIPS) is a valuable tool based on acceptance and commitment to therapy. Psychologically flexible individuals can pursue their goals and values despite experiencing pain. The PIPS was developed to measure inflexibility in patients with chronic pain, regardless of comorbidities, by assessing two distinct domains: avoidance and cognitive fusion.^4^

 In routine clinical assessment, some patients with chronic low back pain have hypomobility of the spine because of stiffness of the postural muscles, making movements that require trunk rotation in activities of daily living difficult.^3^ Therefore, it is necessary to evaluate thoraco-lumbopelvic mobility before, during, and after longitudinal treatment, as it indicates the prognosis of patients through low-cost observation during physical examination.^3^

 Thoraco-lumbo-pelvic mobility can be measured using the leg lateral reach test (LLRT). It was developed by Kim et al.^4^ in healthy patients and adapted by Pontes-Silva et al. for patients with chronic low back pain.^3^ In the test, the patient lies in the supine position, and the rater places a ruler perpendicular to the contralateral knee and measures the maximum distance that the tip of the patient’s foot reaches during thoraco-lumbo-pelvic rotation.^3,4^

 The LLRT is reliable and inexpensive;^3,4^ however, it does not have a cut-off point to diagnose adequate thoraco-lumbo-pelvic mobility, making it just another test whose evaluation generates data that cannot predict prognoses.^5^

## OBJECTIVE

 Under the hypothesis that healthy individuals undergoing the LLRT generate a cut-off point for diagnosing thoraco-lumbopelvic hypomobility, this study aimed to establish a cut-off point for the thoraco-lumbo-pelvic rotation range through the LLRT in patients with chronic low back pain. 

## METHODS

### Design and ethical aspects

 This was a cross-sectional study following the Standards for Reporting Diagnostic Accuracy.^6^ Patients signed an informed consent form before participation. All procedures were approved by the Human Research Ethics Committee of the Universidade Federal do Maranhão (UFMA) (report number: 2.892.673; January 2, 2019). 

### Context

 We publicized the research in the university press and on social networks, including Facebook, WhatsApp, and Instagram (Meta Platforms, Inc., Menlo Park, California, United States) for 12 months (January 2020 to January 2021). Interested parties who contacted the researchers were invited to participate in the recruitment screening. All participants received a verbal explanation of the research procedures and freely agreed to participate in the study. We collected the variables in a private, bright room, with a temperature of 23^°^C and without external noise, in the city of Buriticupu, Maranhão, Brazil. 

### Study size and sampling

 We calculated the a priori sample size using G^*^Power^7^ with a critical t = 1.6675723,^8^ δ = 2.5115915, α = 0.05, and β = 0.80.^9^ As such, the sample required 70 patients divided into two independent and balanced groups (n = 35 + n = 35) whose main prognoses (pain and disability) required a significant difference (P < 0.5)^10^ and size of moderate effect (d value ≥ 0.6).^ 11,12^

### Patients and inclusion criteria

 In the chronic low back pain group (LBPG), we included patients aged 18 to 59 years, of both sexes, who scored ≤ 4 on the Baecke Habitual Physical Activity Questionnaire,^3,13^ with body mass indexes (BMIs) ≤ 26 kg/m^2^,^14^ reports of low back pain for ≥ 90 days,^3^ disability levels ≥ 3^15^ on the Roland-Morris Disability Questionnaire (RMDQ),^16^ and pain levels ≥ 3^15^ points on the Numeric Pain Rating Scale (NPRS).^17^ In the healthy control group (HCG), patients had the same characteristics except for disability and pain. 

 We excluded patients with low back pain attributed to a specific or identifiable cause, such as a history of back surgery or vertebral fractures; spondylosis and spondylolisthesis; presence of radiculopathy or disc herniation confirmed by imaging and physical examination (i.e., changes in sensation, reflexes, or muscle strength); history of physical therapy for low back pain in the past 90 days or medication in the past 7 days; medical diagnosis of cancer, rheumatologic, neurologic, psychiatric, cardiovascular, or metabolic diseases; and pregnancy.^15^

### Assessments and variables

 We measured the iliospinale distance (from the medial malleolus to the anterior superior iliac spine),^18^ weight, height, waist circumference, waist-to-height ratio,^19^ BMI,^14^ C-index,^20^ pain,^17^ disability,^16^ and physical activity.^13^ In addition, patients answered the Pain Self-Efficacy Questionnaire (PSEQ).^21^ All physical examinations and subjective assessments were performed by an independent researcher experienced in the assessment and treatment of chronic low back pain. 

 The NPRS quantifies pain intensity using a sequence of 11 values (0 = no pain; 10 = the worst pain imaginable). Pain intensity was assessed at rest and after movements performed in the LLRT. This scale has been previously validated for this sample.^17^

 The RMDQ was previously validated using a similar sample. This questionnaire is used to measure disabilities in individuals with low back pain. It consists of 24 items describing situations experienced by individuals with low back pain, with scores ranging from 0 to 24 points. The higher the score, the greater the disability.^16^

 The PSEQ consists of 10 items that assess how confident a patient feels in certain situations. Each item has six options with their respective values in ascending order from "not at all confident" to "completely confident." The scores range from 0 to 60, with higher scores indicating higher levels of self-efficacy.^21^

 The Baecke Habitual Physical Activity Questionnaire was also previously validated for this sample and was used to assess the patients’ habitual physical activities.^13^ The instrument uses the domains of work, sport, and leisure to quantify the level of physical activity. The score for each domain ranges from 1 to 5 points, with low scores corresponding to less active patients.^22^

### Leg Lateral Reach Test

 The LLRT measures thoraco-lumbo-pelvic mobility. The patient lies supine on the floor, and the evaluator uses a millimeter ruler perpendicular to the opposite knee on the side being tested to measure the maximum distance that the tip of the foot can reach. Patients moved their feet as far as possible without lifting their shoulders off the floor, rotating only the thoraco-lumbopelvic region.^3,4^ Patients performed three repetitions on each side (right and left); we then calculated the average individual reach distances. 

### Statistical analyses

 We used SPSS software (IBM Corp., SPSS Inc., Armonk, New York, United States, version 17) with an alpha set at 0.05 in all analyses^23^ and checked the distribution of variables using Shapiro-Wilk and Kolmogorov-Smirnov histograms. Categorical variables were compared using Fisher’s exact and chi-squared tests, and quantitative variables were compared using Student’s t-test for unpaired samples (HCG vs. LBPG). Finally, we describe the comparisons in terms of mean, standard deviation, difference between means, confidence interval of the difference (95% CI), and effect size, calculated and classified by Cohen’s d: ≤ 0.2 small effect, 0.5 moderate effect, and ≥ 0.8 large effect ( https://www.psychometrica.de/effect_size.html).^24^

 We used the receiver operating characteristic (ROC) curve to determine the rate of true positives versus false positives at different ranges of motion in the LLRT and found the LLRT cut-off point using the following mathematical model:^25^ (1 – sensitivity)^2^ + (1 – specificity)^2^. The values corresponding to sensitivity and specificity were closest to the point (0,1), which is considered the cut-off point that best differentiates patients with thoraco-lumbopelvic hypomobility from healthy individuals. Furthermore, we used the Youden J index (sensitivity + specificity - 1) to indicate the performance of the identified cut-off point (1 = perfect test; 0 = no diagnostic value)^26^ and the positive (sensitivity/[1 – specificity]) and negative (= [1 – sensitivity]/specificity) likelihood ratios ( http://getthediagnosis.org/calculator.htm).^27^

 Finally, we used logistic regression models adjusted for stature, BMI, iliospinale distance, and waist-to-height ratio to consider the potential influence of these anthropometric variables on the outcome of thoraco-lumbo-pelvic rotation range hypomobility. 

## RESULTS

 The sample consisted of 70 patients divided into two groups (HCG, n = 35; LBPG, n = 35), and was mostly female (HCG = 65.71%; LBPG = 82.85%, P = 0.101), and their characteristics are described in **Table 1**. 

**Table 1:** Characteristics of study participants: Healthy control group (n = 35) and chronic low back pain group (n = 35)

**Variables**		**Groups**	**Minimum**	**Maximum**	**Mean**	**SD**
Age (years)		HCG	19.00	41.00	26.91	5.31
		LBPG	18.00	50.00	31.54	8.84
Waist (cm)		HCG	63.00	90.00	75.09	7.37
		LBPG	62.00	114.00	79.93	13.64
Weight (kg)		HCG	53.10	91.14	68.64	9.32
		LBPG	45.80	134.70	68.38	19.23
Height (m)		HCG	1.50	1.90	1.68	0.09
		LBPG	1.50	1.80	1.61	0.08
Body mass index (kg/m^2^)		HCG	19.00	33.70	24.29	3.37
		LBPG	17.50	46.60	26.03	6.43
Waist-to-height ratio		HCG	0.40	0.50	0.45	0.05
		LBPG	0.40	0.70	0.49	0.08
Conicity index (score)		HCG	0.90	1.20	1.08	0.08
		LBPG	0.90	1.30	1.14	0.09
Pain chronicity (months)		HCG	0.00	0.00	0.00	0.00
		LBPG	3.00	180.00	58.77	46.89
Iliospinale distance Right (cm)		HCG	80.00	96.00	87.80	4.42
		LBPG	74.50	95.00	83.65	5.24
Iliospinale distance Left (cm)		HCG	80.00	96.00	87.91	4.49
		LBPG	74.30	95.00	83.39	5.30
Pain level (NPRS)						
	Rest	HCG	0.00	0.00	0.00	0.00
		LBPG	3.00	10.00	5.34	1.89
	Movement	HCG	0.00	0.00	0.00	0.00
		LBPG	3.00	10.00	5.83	1.85
Disability (RMDQ)		HCG	0.00	0.00	0.00	0.00
		LBPG	3.00	23.00	8.40	4.42
Self-Efficacy (PSEQ)		HCG	0.00	60.00	60	0.00
		LBPG	11.00	60.00	38.57	14.19
Leg Lateral Reach Test (cm)						
	Right	HCG	82.70	110.70	92.59	6.66
		LBPG	24.30	106.30	73.34	18.70
	Left	HCG	67.30	109.30	94.35	7.88
		LBPG	42.70	99.30	74.39	17.23
Physical activity level (Baecke)						
	Occupational	HCG	1.80	3.80	2.53	0.46
		LBPG	1.80	3.80	2.61	0.41
	Sport	HCG	1.00	3.80	2.06	0.70
		LBPG	1.00	3.80	1.98	0.75
	Leisure	HCG	1.50	3.50	2.11	0.54
		LBPG	1.30	3.50	2.04	0.53

SD = standard deviation; HCG = healthy control group; LBPG = low back pain; cm: centimeters; NPRS = Numeric Pain Rating Scale; RMDQ = Roland-Morris Disability Questionnaire; PSEQ = Pain Self-Efficacy Questionnaire.

**Table 2** shows the comparisons of all clinical characteristics of the groups, in which 68.42% of the analyses (13 comparisons) showed a significant difference (P ≤ 0.05) and effect sizes ranging from moderate to large (Cohen’s d ≥ 0.5). 

**Table 2:** Comparisons between groups: Healthy control group (n = 35) and chronic low back pain group (n = 35)

**Variables**		**Groups**	**MD**	**95% CI**	**P value**	**Cohen’s d**
Age (years)		HCG	−4.63	−8.10, −1.15	0.010^*^	0.635^#^
		LBPG				
Waist (cm)		HCG	−4.84	−10.07, 0.39	0.069	0.441
		LBPG				
Weight (kg)		HCG	0.26	−6.95, 7.47	0.943	0.017
		LBPG				
Height (m)		HCG	0.07	0.02, 0.11	0.002^*^	0.822^#^
		LBPG				
Body mass index(kg/m^2^)		HCG	−1.74	−4.19, 0.71	0.160	0.339
		LBPG				
Waist-to-height ratio		HCG	−0.05	−0.08, −0.01	0.006^*^	0.600^#^
		LBPG				
Conicity index (score)		HCG	−0.06	−0.10, −0.02	0.005^*^	0.705^#^
		LBPG				
Pain chronicity (months)		HCG	−58.77	−74.59, −42.95	<0.001^*^	1.773^#^
		LBPG				
Iliospinale distance Right (cm)		HCG	4.15	1.84, 6.46	0.001^*^	0.856^#^
		LBPG				
Iliospinale distance Left (cm)		HCG	4.52	2.17, 6.86	<0.001^*^	0.920^#^
		LBPG				
Pain level (NPRS)						
	Rest	HCG	−5.34	−5.98, −4.70	<0.001^*^	3.996^#^
		LBPG				
	Movement	HCG	−5.82	−5.20, −6.45	<0.001^*^	4.457^#^
		LBPG				
Disability (RMDQ)		HCG	−8.40	−9.99, −6.80	<0.001^*^	2.402^#^
		LBPG				
Self-Efficacy (PSEQ)		HCG	21.43	16.64, 26.22	<0.001^*^	2.136^#^
		LBPG				
Leg Lateral Reach Test (cm)						
	Right	HCG	19.25	12.55, 25.94	<0.001^*^	1.371^#^
		LBPG				
	Left	HCG	19.96	13.57, 26.35	<0.001^*^	1.490^#^
		LBPG				
Physical activity level (Baecke)						
	Occupational	HCG	−0.09	−0.30, 0.12	0.402	0.184
		LBPG				
	Sport	HCG	0.08	−0.26, 0.43	0.645	0.110
		LBPG				
	Leisure	HCG	0.07	-0.18, 0.33	0.578	0.131
		LBPG				

MD = mean difference; CI = confidence interval; HCG = healthy control group; LBPG = low back pain; NPRS = Numeric Pain Rating Scale; RMDQ = Roland-Morris Disability Questionnaire; PSEQ = Pain Self-Efficacy Questionnaire.

* Significant difference (t-test, P ≤ 0.05)

# Moderate effect size (Cohen’s d ≥ 0.5)

**Figure 1:**
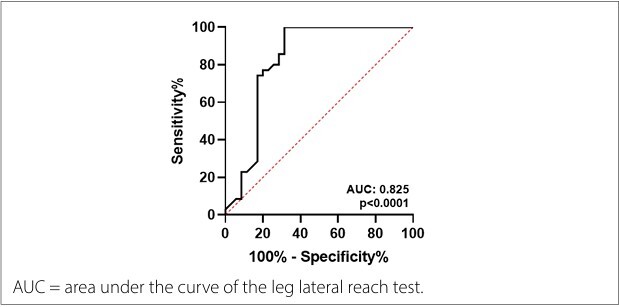
Receiver Operating Characteristic (ROC) curve.

 According to the ROC curve analysis, the cut-off values that produced ideal sensitivity and specificity were ≤ 82.85 cm during LLRT (sensitivity = 0.97, specificity = 0.31). The area under the curve, Youden’s J index, and positive and negative likelihood ratios are presented in **Table 3** and **Figure 1**. 

**Table 3:** Cut-off value, sensitivity, and specificity of thoraco-lumbar-pelvic mobility (cm) based on the leg lateral reach test; cm: centimeter

**Variable**	**Cutt-of**	**Sensitivity**	**Specificity**	**AUC 95% CI**	**Youden Index**	**Positive Likelihood**	**Negative Likelihood**
LLRT (cm)	≤ 82.85 (cm)	0.97	0.31	0.826 (0.717, 0.934)	0.66	1.41	0.10

AUC = area under the curve; CI = confidence interval; LLRT = leg lateral reach test.

 The logistic regression model results showed that the effect of the LLRT remained statistically significant after adjustment (P < 0.05), with an estimated odds ratio (OR) of approximately 0.5. The following anthropometric variables were not significantly associated with thoraco-lumbo-pelvic rotation range hypomobility: height (P = 0.700923, OR = 0.024421), BMI (P = 0.231799, OR = 0.752938), waist-to-height ratio (P = 0.875384, OR = 11.758806), or iliospinale distance (P = 0.646721, OR = 0.927256). Therefore, these findings suggest that anthropometric variables do not confound the association between the LLRT and thoraco-lumbo-pelvic rotation range hypomobility. 

## DISCUSSION

### Main results synthesis

 Our study confirmed the hypothesis and established a cut-off point for the diagnosis of thoraco-lumbo-pelvic rotation range hypomobility in patients with chronic low back pain (LLRT ≤ 82.85 cm). Additionally, the main outcomes (pain and disability) showed a significant difference (P ≤ 0.05) and an effect size ranging from moderate to large (Cohen’s d ≥ 0.5) according to the a priori sampling requirement. 

### Study strengths

 This is the first study to propose a cut-off point for the LLRT. Sensitivity and specificity values showed that the LLRT has an excellent ability to diagnose thoraco-lumbo-pelvic rotational range hypomobility in patients with chronic low back pain. However, we recommend that researchers and clinicians observe the biomechanical and anthropometric characteristics of patients undergoing LLRT and compare them with those of our sample, because they are known to be associated with lumbar function.^28^

 Kim et al. found excellent reliability for the LLRT in a healthy sample with a mean reach of 73.19 cm, but they did not mention the need for a cut-off point for the LLRT.^4^ In addition, the authors did not publish subsequent articles using the test itself.^4^ Our results confirm the significant difference and large effect size (P < 0.001, d ≥ 0.5) between healthy patients and patients with chronic low back pain undergoing the LLRT, highlighting the importance of this point omitted by the pioneers (i.e., Kim et al.).^4^

 The second article on the LLRT was published by Pontes-Silva et al. using a sample with chronic low back pain.^3^ They also found excellent reliability and a similar mean range (73.29 cm), but they omitted the validation of a cut-off point for the diagnosis of thoraco-lumbo-pelvic rotation range hypomobility.^3^ Our study has a sample similar to the recent one in all clinical and anthropometric variables; therefore, our cut-off point (≤ 82.85 cm) is in agreement with the previously tested reliability.^3^

### Clinical applicability of outcomes obtained

 Translating scientific findings into clinical practice is one of the greatest challenges in research, as reproducing methods tested in laboratories with the infrastructure to do so is not feasible for most healthcare professionals. Thus, the LLRT has excellent discriminatory power to diagnose thoraco-lumbo-pelvic rotation hypomobility in patients with chronic low back pain and presents itself as an assessment tool with potential for application because it is simple, fast, accessible, and does not require a large physical space (which may even be used for home care).^3,4^

 Practice guidelines for the management of musculoskeletal pain recommend thorough physical examination and outcome measures to monitor prognosis.^29^ Therefore, considering that patients with chronic low back pain may present with asymmetries in trunk rotation and decreased spinal flexibility,^30^ the inclusion of the LLRT in clinical and experimental settings may guide therapeutic approaches to bilaterally compare and monitor the mobility of thoraco-lumbo-pelvic rotation in this population.^4^

 The low specificity of the LLRT results in a high rate of false positives, indicating that individuals without true hypomobility may be incorrectly identified as having hypomobility. In clinical decision-making, this can lead to overtreatment, unnecessary interventions, or the misallocation of resources. Although the LLRT may be useful as an initial screening tool owing to its sensitivity and ease of application, its results should be interpreted with caution. 

 Therefore, to optimize its use, the LLRT should not be used in isolation. It is best employed in combination with other diagnostic tools such as more specific physical tests, imaging when indicated, or clinical judgment based on patient history and presentation. This multimodal approach balances the tradeoffs between sensitivity and specificity, improves diagnostic accuracy, and ensures appropriate clinical decisions. 

### Limitations and prospects for new studies

 We are aware that the biostatistics of such studies are based on measures of central tendency to describe and/or infer the analyzed results. As such, we emphasize that the distance achieved in the LLRT may be influenced by stature and length of the low limb, taking into account the biological individuality of the patients (i.e., individual patient data).^31^ Therefore, before using the cut-off point established in this study, we suggest that clinicians and scientists check whether there is a significant difference (> 5%) between their patients and those in our sample for the aforementioned characteristics. 

 Although the LLRT showed high sensitivity (97%), which is favorable for identifying at-risk individuals, its specificity was low (31%), indicating a high false positive rate. These results suggest that, while the test effectively rules out individuals without reduced thoraco-lumbo-pelvic mobility, it may also incorrectly classify many individuals as positive, even when they are not clinically impaired. This reduces its usefulness for confirming a diagnosis. 

 Several factors may account for the low specificity observed in this study. For example, LLRT performance may overlap between individuals with and without functional impairments, especially in heterogeneous populations. This overlap could result from variability in physical activity levels, compensation strategies, or differences in trunk and low limb biomechanics. In addition, anthropometric characteristics, such as stature, leg length, and pelvic width, may influence the reach distance during the LLRT and potentially bias the classification threshold. 

 Future studies should stratify the cut-off points based on height or other anthropometric measures. This would increase specificity without substantially compromising sensitivity, provided that robust samples were used. In addition, combining the LLRT with other functional tests or clinical indicators could improve overall diagnostic accuracy. Despite these limitations, the high area under the ROC curve (area under the curve = 0.826) and low negative likelihood ratio indicate that the LLRT holds promise as a screening tool for ruling out clinically significant dysfunction, but not as a standalone diagnostic measure. 

## CONCLUSION

 Patients with chronic low back pain and an LLRT range ≤ 82.85 cm have hypomobility in the thoraco-lumbo-pelvic rotation range. 

## Data Availability

The data that support the findings of this study are available from the corresponding author, André Pontes-Silva, upon reasonable request.
